# Ebola virus disease contact tracing activities, lessons learned and best practices during the Duport Road outbreak in Monrovia, Liberia, November 2015

**DOI:** 10.1371/journal.pntd.0005597

**Published:** 2017-06-02

**Authors:** Caitlin M. Wolfe, Esther L. Hamblion, Jacqueline Schulte, Parker Williams, Augustine Koryon, Jonathan Enders, Varlee Sanor, Yatta Wapoe, Dash Kwayon, David J. Blackley, Anthony S. Laney, Emily J. Weston, Emily K. Dokubo, Gloria Davies-Wayne, Annika Wendland, Valerie T. S. Daw, Mehboob Badini, Peter Clement, Nuha Mahmoud, Desmond Williams, Alex Gasasira, Tolbert G. Nyenswah, Mosoka Fallah

**Affiliations:** 1 Ebola Response Team, World Health Organization, Monrovia, Liberia; 2 Ebola Response Team, Action contre la Faim, Monrovia, Liberia; 3 Ebola Response Team, International Rescue Committee, Monrovia, Liberia; 4 Ebola Response Team, Liberia Ministry of Health, Monrovia, Liberia; 5 Ebola Response Team, United States Centers for Disease Control and Prevention, Monrovia, Liberia; Santa Fe Institute, UNITED STATES

## Abstract

**Background:**

Contact tracing is one of the key response activities necessary for halting Ebola Virus Disease (EVD) transmission. Key elements of contact tracing include identification of persons who have been in contact with confirmed EVD cases and careful monitoring for EVD symptoms, but the details of implementation likely influence their effectiveness. In November 2015, several months after a major Ebola outbreak was controlled in Liberia, three members of a family were confirmed positive for EVD in the Duport Road area of Monrovia. The cluster provided an opportunity to implement and evaluate modified approaches to contact tracing.

**Methods:**

The approaches employed for improved contact tracing included classification and risk-based management of identified contacts (including facility based isolation of some high risk contacts, provision of support to persons being monitored, and school-based surveillance for some persons with potential exposure but not listed as contacts), use of phone records to help locate missing contacts, and modifications to data management tools. We recorded details about the implementation of these approaches, report the overall outcomes of the contact tracing efforts and the challenges encountered, and provide recommendations for management of future outbreaks.

**Results:**

165 contacts were identified (with over 150 identified within 48 hours of confirmation of the EVD cases) and all initially missing contacts were located. Contacts were closely monitored and promptly tested if symptomatic; no contacts developed disease. Encountered challenges related to knowledge gaps among contact tracing staff, data management, and coordination of contact tracing activities with efforts to offer Ebola vaccine.

**Conclusions:**

The Duport Road EVD cluster was promptly controlled. Missing contacts were effectively identified, and identified contacts were effectively monitored and rapidly tested. There is a persistent risk of EVD reemergence in Liberia; the experience controlling each cluster can help inform future Ebola control efforts in Liberia and elsewhere.

## Author summary

Contact tracing is one of the key response actions necessary for controlling spread of Ebola Virus Disease (EVD). Contact tracing is comprised of several different activities: identification of persons who have been in contact with confirmed EVD cases, close monitoring contacts for EVD symptoms, and management of symptomatic persons. Closely monitoring contacts of confirmed EVD cases allows for the rapid identification of symptomatic individuals, which in turn facilitates early testing, medical intervention, and isolation of new cases. This reduces the possibility of the continued spread of the virus within communities. Delayed and ineffective contact tracing contributed to the extensive transmission of EVD during the 2014–2015 outbreak in West Africa. Clusters of EVD reemergence are likely to occur, therefore understanding and addressing the challenges of implementing and managing contact tracing remains essential to halting transmission and minimizing morbidity and mortality associated with EVD. This paper assessed the contact tracing activities in response to EVD reemergence to identify best practices for responses to future Ebola clusters. The work is also applicable to contact tracing for other infectious diseases.

## Introduction

The largest outbreak of Ebola Virus Disease (EVD) on record began in Guinea in 2013 and spread to Liberia by March 2014 [1,2]. With the support of partner agencies and organizations, the Liberian Ministry of Health (MOH) directed the implementation of multiple interventions that led to control of the initial epidemic [3] and two subsequent EVD clusters [4,5]. After a previous declaration several months before [6], Liberia was declared free of EVD transmission for a second time on September 3, 2015 [7].

Contact tracing, a cornerstone intervention to halt transmission of infection, is the process of identifying, assessing, and monitoring people who may have been exposed to a disease to prevent onward transmission [8,9]. The recommended practice to control Ebola outbreaks is to identify contacts of confirmed EVD cases and systematically monitor them twice daily for 21 days from their most recent potential exposure to an infectious case [9]. This allows for the rapid identification of people who become symptomatic and facilitates early isolation and treatment to prevent further transmission [10,11]. While contact tracing was critical to control of the main Ebola outbreak and subsequent clusters, several challenges were encountered. These included difficulty locating contacts, difficulty with contacts completing 21 days of monitoring and unwillingness of symptomatic contacts to attend an Ebola Treatment Unit (ETU) be tested for Ebola among others.

Following the second declaration of no active EVD transmission in Liberia, the country maintained heightened EVD surveillance [10]. On November 19, 2015, a 15-year-old boy with symptoms compatible with Ebola was seen at a health care facility in Monrovia, in Montserrado County, Liberia, resulting in an alert to public health authorities. He was isolated and subsequently confirmed to have EVD [12]. The Liberia Incident Management System (IMS) was immediately activated to respond. Since the index patient was from the Duport Road area of Monrovia, the cluster was referred to as the “Duport Road Cluster”. The confirmed case and family members residing in the same household were transferred to an ETU. Two of these family members were confirmed to have EVD. The response team immediately initiated identification and monitoring of contacts, incorporating adaptations to previous approaches that aimed to improve the completeness and effectiveness of these activities. As this was the first cluster response in Liberia to incorporate administration of Ebola vaccination to identified contacts and contacts of contacts, procedures for monitoring vaccinated contacts were developed.

On March 29 2016, WHO declared the Ebola Public Health Emergency of International Concern (PHEIC) over but recognized new clusters due to reemergence had occurred and are likely to continue to occur. Thus, countries must maintain the capacity and readiness to prevent, detect, and respond to any new cases or clusters [13]. We describe the approaches to contact tracing during the response to the Duport Road Cluster, and outcomes of these activities, to inform future Ebola control efforts.

## Methods

Adaptations to previously used contact tracing procedures included: 1) classification of contacts by risk status and differential management depending on risk status, 2) use of phone records to identify missing contacts, and 3) use of modified data collection and display tools. The detailed components of contact tracing employed during this cluster are outlined in Table 1.

**Table 1:** Contact tracing components, challenges, and solutions from the Duport Road EVD outbreak, November–December, 2015.

Step	Description	Challenges	Solutions
**1. Contact identification**	Identify persons who may have come in contact with a symptomatic EVD case.	Case may be deceased or unable to speak with investigators due to illness.	Conduct interviews with family, friends, neighbors to determine who may have been in contact with the case while symptomatic.
	Conduct interviews with potential contacts to determine if identified persons are actual contacts.		
	Locate all possible contacts for further evaluation.	Identified contacts may attempt to hide from contact tracing teams due to fear or stigma.	Ask local family members, community members, or community leaders where missing contacts may be hiding after explaining the importance of daily monitoring.
			If contacts have fled to neighboring areas, coordinate with local government and surveillance officials.
			If still unable to locate missing contacts, government officials may consider issuing a subpoena to local phone companies to determine contacts' whereabouts.
		Families or communities may purposely not identify persons who came into contact with symptomatic case due to fear or stigma.	Continue asking family and community members throughout follow up period about any other persons who may have come in contact with symptomatic case. Once trust is established with assigned contact tracer, they may be willing to identify others at risk.
		Individuals who actually did not come into contact with a symptomatic case may be dishonest about their potential exposures: for example, if those listed as contacts receive benefits such as food and water rations due to in-home isolation.	Ideally contacts should be identified before any information on in-home isolation and any resulting benefits packages are discussed. If individuals are later identified as a) not true contacts or b) contacts of a suspected case who subsequently was determined not to have the disease after laboratory testing, they should cease being monitored.
**2. Contact listing**	List as contacts individuals that: 1) touched body fluids of case 2) had direct physical contact with body of case 3) slept or ate in same household as case 4) manipulated clothing of or shared linens with case 5) had a close interaction with case that did not involve physical contact	Potential contacts may be unwilling to disclose any kinds of exposures due to fear or stigma.	Err on the side of caution and assume they are a higher risk classification. As assigned tracers develop relationships with the contacts and earn their trust, re-evaluate exposure type and resulting risk classification as more information becomes available.
	Classify risk status of identified contacts.		
	Tactfully and politely inform contacts of their risk and status, and explain the purpose of contact tracing and the monitoring procedure so contacts understand what is happening.	Identified contacts may attempt to hide from contact tracing teams due to fear or stigma.	Ask local family members or community members where missing contacts may be hiding after explaining the importance of daily monitoring.
			If contacts have fled to neighboring areas, coordinate with local government and surveillance officials.
			If still unable to locate missing contacts, government officials may consider issuing a subpoena to local phone companies to determine contacts' whereabouts.
		Contacts may resist in-home isolation or isolation at another facility due to fear, stigma, fear or losing their jobs, or fear or academic penalty.	Contact tracers and supervisors should explain the importance of contact tracing, the support that will be provided to contacts under monitoring, and government officials should provide documentation to contacts' employers or academic institutions explaining the situation and public health importance of contact tracing, excusing contacts from any penalties or repercussions from complying with isolation and monitoring.
	Identify trained local contact tracers to monitor all contacts.	Communities or counties may not have maintained a record of trained contact tracers	Work with local NGOs and other organizations to identify community health workers or others who have training in contact tracing
	Assign contacts to each contact tracer.		If none available, identify available community health workers and establish training sessions as soon as possible. Trainings should focus how to use the thermometers, symptoms to look for, correct completion of daily monitoring form, potential issues, and how to engage politely with contacts Supervisors should be prepared to assume contact tracing duties until enough trained contact tracers are available.
**3. Daily monitoring**	Contac tracers visit each of their assigned contacts twice daily.	Contacts refuse to interact with tracer.	Contact tracer must alert supervisor, and the supervisor should visit any resistant contacts. If necessary, consider engaging with community support groups or social mobilization teams. If unable to assess contact, this information must be noted on the daily monitoring form.
	During each visit, contact tracers take and record the temperature of each contact and visually observe them for signs of disease.	Tracers record invalid temperatures for contacts.	This is likely a training issue. Supervisors should review all information obtained and submitted by contact tracers to ensure it is accurate. If issues like invalid body temperatures arise, refresher trainings for contact tracers or greater supervision may be required.
		Contact develops symptoms consistent with disease.	Contact tracer must alert supervisor immediately.
**4. Supervision of contact tracing**	Contact tracers report the health status of each contact to their supervisors each day.	Contact develops symptoms consistent with disease.	Supervisor assesses contact. If symptoms are consistent with disease, contact should be transported to ETU. Contacts must have 2 negative tests at least 48 hours apart to be determined free of disease
			If available, consider using rapid diagnostic test performed by mobile laboratory personnel at contact's home or place of isolation.
			If a vaccination campaign has been launched as part of the outbreak response, surveillance teams should liaise with vaccination campaign personnel to determine if symptomatic contacts received a vaccine. Possible vaccination side effects include fever so it is important to determine if symptoms are from receiving the vaccination or due to developing disease.
	Supervisors routinely monitor contact tracing activities and perform spot checks or quality checks on home visits.	Information pertaining to daily monitoring is not collected or documented properly.	Supervisors must address these issues with contact tracers through refresher trainings, field supervisory visits, or replacement of contact tracers if repeated poor performances.
**5. Discharge of contacts**	Contact tracers must assess all contacts twice on day 21.	Contact(s) not seen or only seen once on 21st day.	Tracers must visit contacts again on 22nd day in order to determine they are still free of signs/symptoms of disease before contacts can be discharged from monitoring.
	Reintegrate contacts back into the community.	Community may not accept contacts or ostracize them out of fear or stigma.	Consider engaging the community (especially community and faith leaders) in a 'graduation ceremony' to fully reintegrate contacts after monitoring and show the community that contacts no longer posed any risk.
**6. Data management**	Contact tracers complete daily monitoring forms and turn into supervisor.	Information pertaining to daily monitoring is not collected or documented properly.	Supervisors must address these issues with contact tracers through refresher trainings, field supervisory visits, or replacement of contact tracers if repeated poor performances.
	Supervisor reviews all daily monitoring forms for errors or signs/symptoms of concern at the end of each day.		
		Symptomatic contacts may be identified.	At this point in the day, supervisors should have already been alerted about symptomatic contacts and arranged for testing. If this has not already been done, the supervisor and surveillance team must do so now. Additionally, this data must be entered into the summary data sent to IMS, the Dashboard, and the master list.
	Conduct evening feedback sessions for all supervisors and district surveillance team to discuss and address any issues or challenges.	Supervisors may not be able to attend meeting.	Supervisors should alert surveillance team of their delay and provide update on any missing or symptomatic contacts as well as any challenges from that day over the phone.
	Collect the summary data containing the total number of contacts listed that day along with: 1) number of contacts seen/monitored that day 2) number of contacts not seen/monitored that day 3) number of missing contacts 4) number of contacts lost to follow up (missing for >3 days) 5) number of symptomatic contacts 6) challenges encountered with contact tracing activities.	Missing data or lack of data collection and management knowledge/skills among contact tracing and/or surveillance team staff.	If issues due to missing data, data management and surveillance team should work with contact tracing teams (supervisors and tracers) to identify any issues in collecting the required information.
			If issues due to lack of knowledge or skills relating to data collection among contact tracing or surveillance teams, supporting partners may need to step in to streamline data collection and analysis process in order to ensure the information collected reflects an accurate representation of the current situation.
	Share summary data with surveillance and IMS personnel daily.		
	Enter each contact into master list and update daily.		
	Enter each contact into Dashboard (see Fig 2) and update daily.		

### Identification and management of contacts

#### Contact identification and classification

Case investigators carefully interviewed the family to identify other persons the cases may have come in contact with while symptomatic. The IMS case management team met with each health care worker (HCW) contact to discuss their possible workplace exposures. Case investigators, supported by contact tracers, evaluated possible community contacts. Individuals were listed as contacts if they 1) touched body fluids of a case, 2) had direct physical contact with the body of the case, 3) slept or ate in the same household as the case, 4) manipulated the clothing of or shared linens with the case or 5) had a close interaction with a case that did not involve physical contact. Persons whose only interaction with a case was co-attendance at a school were evaluated but were not classified as contacts unless a specific exposure was identified.

Contacts were classified by risk status. Those who had direct contact with cases or their body fluids were designated high risk (criteria 1 and 2 above) unless it was determined without doubt that appropriate personal protective equipment (PPE) had been used. Those who did not have direct contact with the case or their body fluids (criteria 3–5 above) or who did but always used appropriate PPE were designated as low risk. The list of contacts, their location, and risk status were recorded on the contact listing form. The Montserrado County Health Team (MCHT) data manager entered the details from this form into the contact tracing database.

#### Contact tracing and daily monitoring

**Fig 1:**
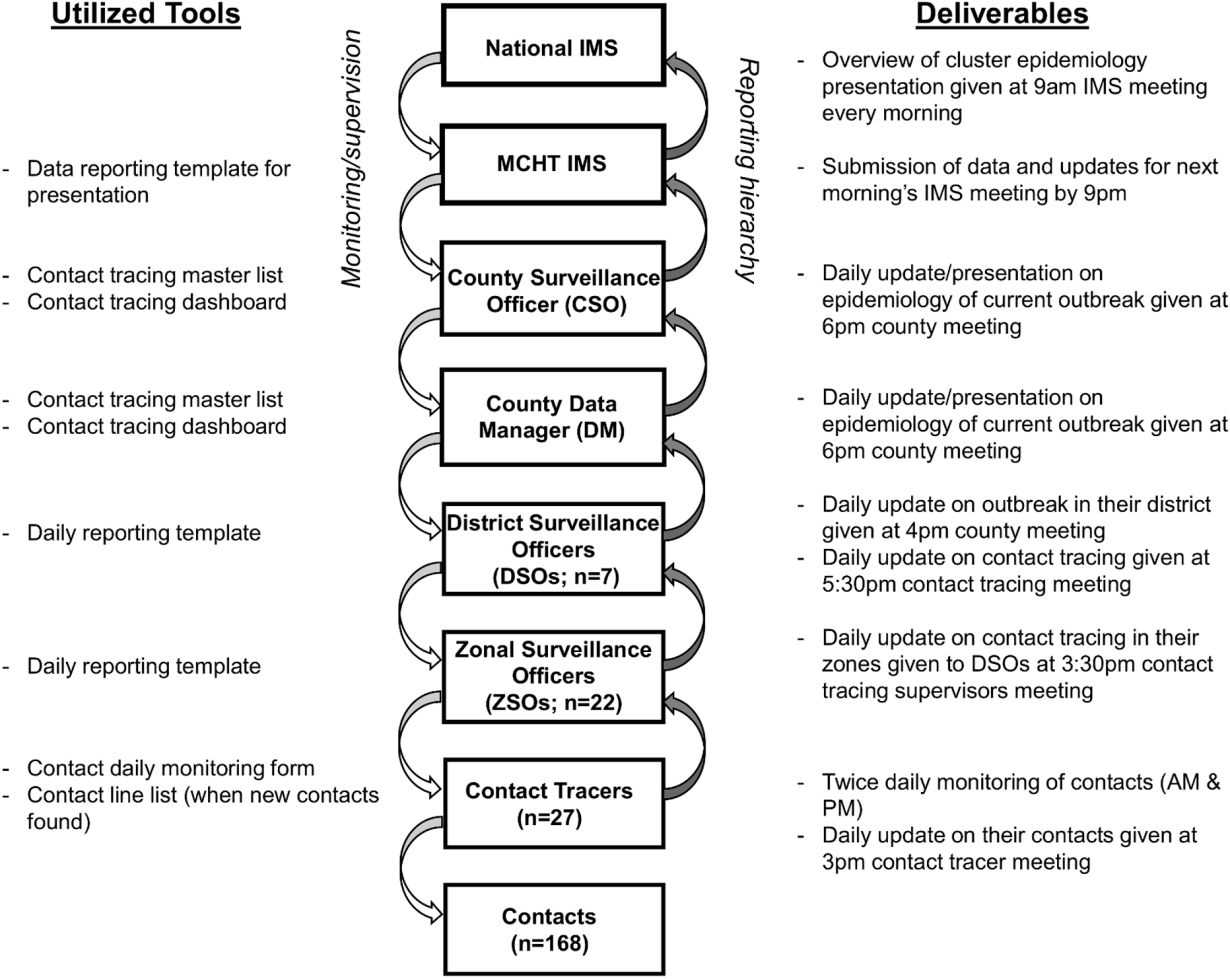
Montserrado County EVD contact tracing structure and information flow for Duport Road outbreak, November–December, 2015.

The County Health Officer (CHO) activated contact tracing teams and provided team members with refresher training. Montserrado County consists of seven districts (only four were affected), each with a District Surveillance Officer (DSO), and 22 zones, each with a Zonal Surveillance Officer (ZSO). The ZSOs supervised contact tracing within their zones (Fig 1). Each contact tracer (n = 27) was assigned 6–16 persons, covering 1–3 households depending on the number of contacts in each. Throughout the response, experienced mentors provided additional on-the-job training and recommendations to the contact tracing teams.

Contact tracers and supervisors located the contacts, introduced themselves, and explained the purpose of contact tracing and what to expect for the remainder of the 21-day period. The IMS requested that all contacts remain at their homes in order to facilitate monitoring and prevent further EVD transmission. Health care workers who were deemed to be high-risk contacts as a result of a workplace exposure were housed in a Guest House and away from their families who were not at risk. For 21 days from the last date of exposure to a confirmed case, a trained contact tracer visited each contact twice daily (early morning and late afternoon). The contact tracers obtained each contact’s temperature, inquired about his/her health, and screened for possible EVD symptoms, which were all recorded on the contact monitoring sheet. Contact tracers were trained to develop a rapport with contacts to collect further information as it became available, including about additional contacts not listed during the case investigation.

#### Support for quarantined contacts

The in-home quarantine of contacts increased their need for further supportive provisions. Partner agencies provided food and water to contacts' homes and psychosocial teams made regular visits to provide any support needed with regard to individual’s mental health and the psychosocial effects of quarantine. The MOH provided written documentation to employers, enabling contacts to abide by mandatory quarantines without fear of losing their jobs. Once the outbreak concluded, MOH provided additional documentation to contacts’ employers stating they could return to their jobs with no risk to the community. When quarantined persons were students, the MOH also provided written documentation to schools, including universities, excusing their students from exams to ensure they remained in quarantine without academic penalty.

To fully reintegrate contacts back into the community after their 21-day quarantine, promote community unity, and to show the community that contacts no longer posed any risk, a “graduation ceremony” was held where contacts were formally discharged from monitoring and given back to the community.

#### School–based surveillance

Two of the identified cases had attended school while symptomatic. While people whose only known interaction with the cases was co-attendance at the school were not listed as contacts, school based surveillance was established. Contact tracers assigned to the school recorded the attendance and temperature of students each school day during the monitoring period. In addition, community based active case finders went door to door to identify any students that may have been ill and not attending school. On weekends when school was not in session, contact tracers visited the community to check on the students but did not routinely measure temperatures for all students. The contact tracers reported to their supervising ZSOs daily, providing enrollment numbers and monitoring summaries to the MCHT data team.

#### Management of symptomatic contacts

If a contact tracer determined a contact to have symptoms compatible with EVD, the ZSO was called to investigate and verify the report. If the case definition was fulfilled, the contact was transferred to the ETU for further evaluation, close monitoring, and EVD testing. If a contact had any symptoms consistent with possible EVD, but did not fulfill the case definition, the ZSO alerted laboratory personnel who were dispatched to the contact’s household for field blood sample collection. If the contact tested negative and was no longer symptomatic, he/she remained isolated at home while awaiting a second negative blood test 48 hours after the first [14, 15]. When symptoms were deemed likely unrelated to EVD but persistent after the first negative test, the contact was transferred to an isolation unit in a healthcare facility until a second blood test was negative.

#### Management of vaccinated contacts

As part of the Duport Road EVD outbreak response, the MOH implemented vaccination of persons at high risk of infection to prevent further spread of infection. Contact tracers were informed about the Ebola vaccination strategy including procedures for management of vaccinated contacts who developed a fever or other EVD symptoms. Laboratory testing for EVD was conducted among vaccine recipients if they became symptomatic during the 21-day monitoring period since symptoms could have been due to EVD rather than vaccine side effects.

### Identification of missing contacts

Active case finding teams conducted interviews with families, neighbors, employers and co-workers of cases to locate missing contacts. For contacts that had left Montserrado County, the County Health Teams in the respective areas worked with the Montserrado County Team to assist in finding the missing contacts through the established surveillance system. Reluctance to provide locating information and unwillingness of some contacts to be monitored led the MOH to subpoena mobile phone companies in order to use phone records to track down missing contacts. The teams used the phone records to determine the communities and locations where previous calls were made or text messages sent and received, and conducted house-to-house searches around the various communities to find missing contacts. The MOH arranged return transportation for contacts who left Montserrado County before tracing efforts began.

### Enhanced data management procedures

The ZSOs reviewed the contact monitoring forms and used them to complete a daily summary that was reported to the DSOs. The DSOs, in turn, used this information to compile a district summary form and report to the county data manager each evening. A contact tracing feedback session was held at 6pm daily for all DSOs, ZSOs, and contact tracers as needed. An aggregated summary table, including tracers, contacts, and contact status (number monitored/not monitored, lost to follow up, or missing) was created and updated during the nightly feedback session together with the contact tracing database. Daily descriptive analysis was conducted on contact tracing activities and these data, in conjunction with case data, were presented during the IMS meeting the following morning (Fig 1).

**Fig 2:**
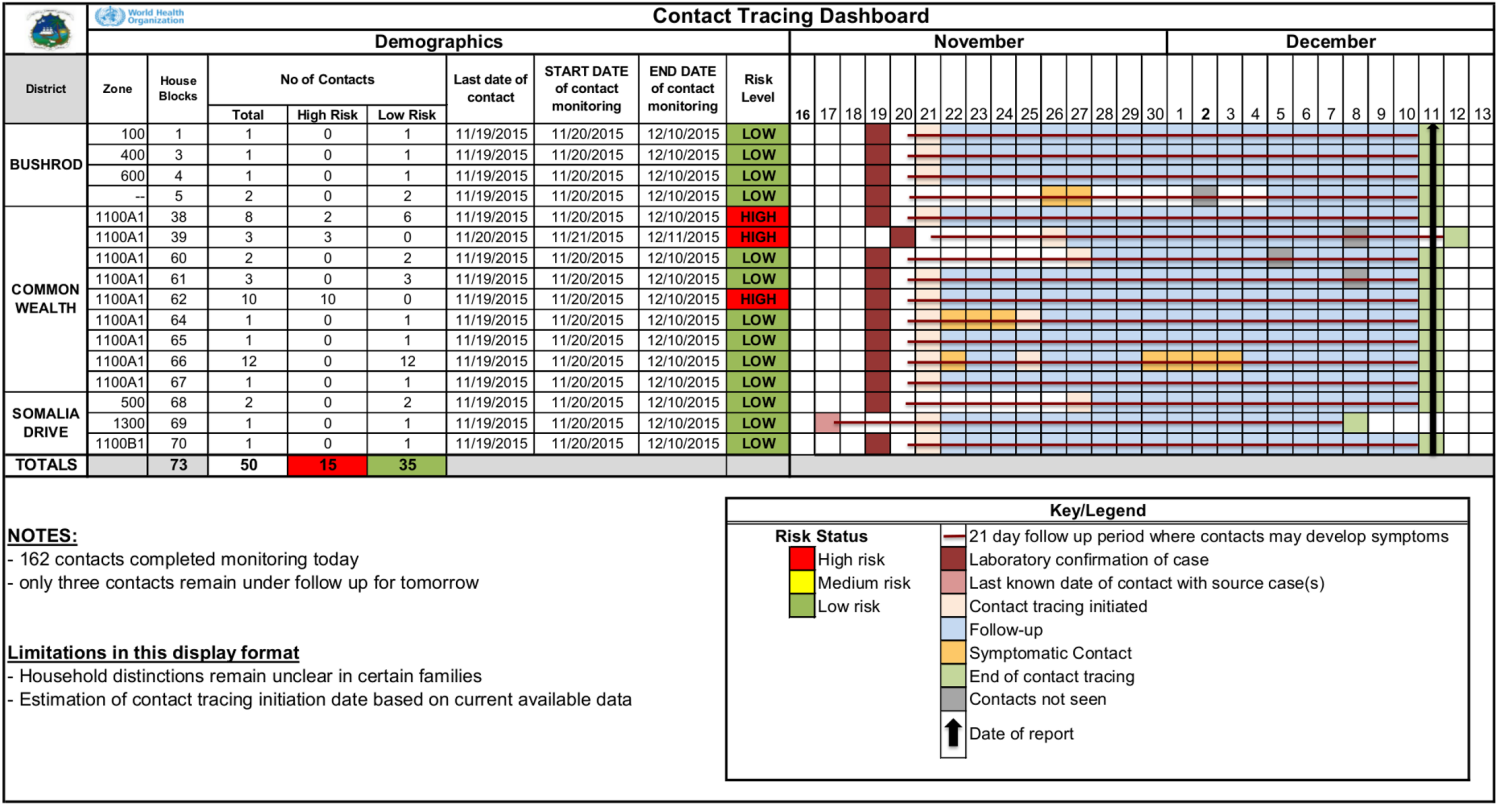
Sample contact tracing dashboard (de-identified) used in the Duport Road EVD outbreak, Montserrado County, November–December 2015.

A dynamic contact tracing dashboard was created to enable tracking of contacts over time by household, using data collected from the field. Contacts were grouped by household, with the head of household’s name listed on the dashboard. The total number of contacts, contacts by risk status, and overall risk status of the household were displayed. The dashboard provided the name of the contact tracer, supervising DSO, district, and zone and allowed for visualization of the 21-day follow-up period of all contacts. Color coding indicated the last date of possible exposure from which the 21-day follow up period started, the date contact tracing was initiated, successful daily follow-up, dates contacts were not seen, dates any contacts were symptomatic, and the last date of contact tracing. A moveable arrow bar indicated the date/day of follow-up (Fig 2).

Lastly, a master list of all contacts was maintained in Microsoft Excel, and was updated and resaved with new corresponding data each evening.

## Results

### Identification and management of contacts

#### Contact tracing teams

There was a two-day delay in implementation of contact tracing activities due to challenges related to identifying individuals to undertake the contact tracing (no register was available of those who had previously done the role), the need for refresher training, job aides and no standard approach to ensuring the availability of required equipment and supplies (e.g. thermometers, scratch cards for communication, rain jackets and boots, and funds to cover transport costs). Surveillance technical experts identified knowledge gaps among contact tracers at the beginning of the outbreak during their field supportive supervision activities. Gaps related to knowledge of the acceptable temperature range, proper use of medical infrared thermometers, and the difference between contacts and contacts-of-contacts. There was also confusion regarding transmission of EVD and fear among some contact tracers, resulting in the unnecessary use of PPE.

#### Management of contacts

Early in the response, there were delays in the delivery of food and water to contacts under quarantine as well as multiple visits to contacts’ homes by different teams daily. This resulted in confusion and frustration among contacts and threats to disobey the quarantine restrictions. The problem with the multiple visits was raised a number of times at the daily IMS meeting and it was requested that all those who visit a contact’s home inform the contact tracers so they could manage the visits. This model was agreed by the IMS chair and followed for the remainder of the monitoring period. Contacts’ privacy was invaded by journalists taking pictures outside their homes, leading to further threats to leave due to the stigmatization of being publically identified as a contact.

#### Demographics of contacts

**Fig 3:**
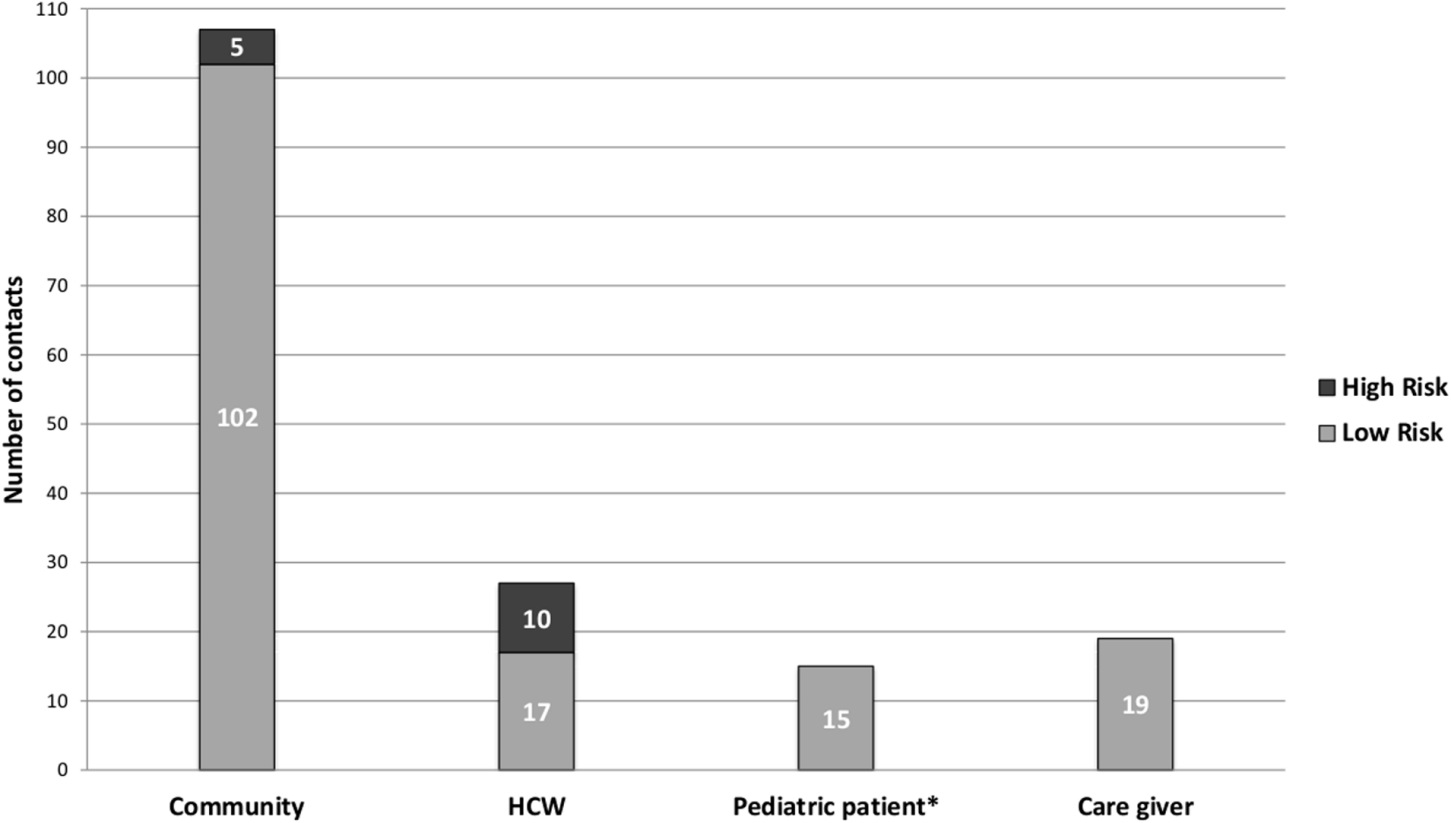
Contacts by risk status, Duport Road EVD outbreak, Montserrado County, November–December 2015. *Includes two pediatric patient contacts who died during the monitoring period due to underlying illnesses.

**Fig 4:**
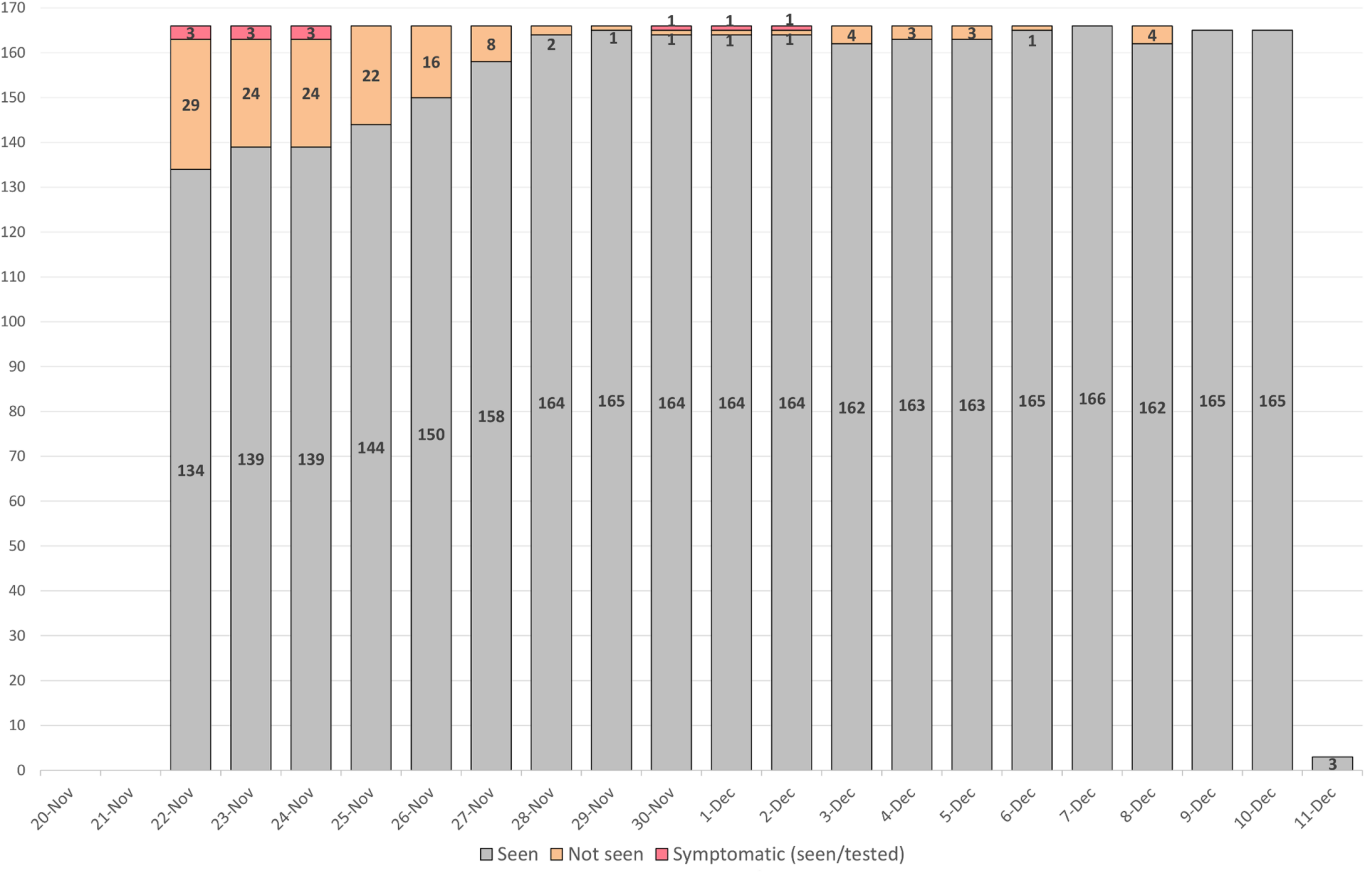
Contact status by day of monitoring, Duport Road EVD outbreak, Montserrado County, November–December 2015.

A total of 168 contacts associated with the three confirmed cases were found among 73 households across 4 districts in Montserrado County; 68% (n = 115) were in Commonwealth District. Forty-five percent of contacts were male; ages of the contacts ranges from 2 weeks to 72 years with a median age of 14 years although data on age was missing for 42.9% (72 of 168) contacts. The contacts were primarily members of the community where the family of the alert case lived (64%; n = 107), (Table 2; Fig 3). The status of contacts by day of monitoring is shown in Fig 4.

**Table 2:** Contact tracing results from the Duport Road EVD outbreak, November–December, 2015. This table displays the summary information for the 168 contacts monitored in response to the Duport Road EVD outbreak.

Contact data breakdown		Number	Percentage
Total number of contacts		168	—
	Community	107	64%
	Health care worker	27	16%
	Pediatric patient	15	9%
	Care giver of pediatric patient	19	11%
Total number of households		73	—
Total high risk contacts		15	9% (of total)
	Community	5	33%
	Health care worker	10	67%
	Pediatric patient^	0	0%
	Care giver of pediatric patient^	0	0%
Total low risk contacts		153	91% (of total)
	Community	102	67%
	Health care worker	17	11%
	Pediatric patient^	15	10%
	Care giver of pediatric patient^	19	12%
Total symptomatic contacts		11^✝^	7% (of total)
	Transferred to ETU	3	27%
	Field blood draw	8	73%
Contact deaths unrelated to EVD		2	1%

^pediatric patients and caregivers of the pediatric patients at the hospital where the alert case presented

^✝^One contact was ill but with symptoms not consistent with EVD and a history of prior illness

Nine percent (n = 15) of contacts were classified as high risk, of which 67% (n = 10) were HCWs quarantined in a guest house (Fig 3).

#### Symptomatic contacts

Three contacts developed symptoms consistent with EVD and were taken to the ETU for laboratory testing and supportive care; all tested negative for EVD. Eight other contacts developed at least one symptom consistent with EVD, but did not fulfill the EVD suspect case definition, so a blood draw was performed at home and tested. All of these individuals tested negative for EVD.

One contact developed non-EVD related symptoms and had a history of previous illness. An initial blood sample was collected at home and tested negative for EVD. Because the symptoms worsened, the contact was transferred to a nearby health facility with an isolation unit for treatment while awaiting a second negative blood test. This contact was not taken to an ETU.

Two contacts died from causes other than EVD during the monitoring period (both tested negative twice for EVD). They were both patients that had been admitted to the pediatric ward at the time the alert case was symptomatic and both had other underlying illnesses.

The number of contacts monitored each day changed as contacts died or completed 21 days of follow-up from their last possible date of exposure. The last three remaining contacts under monitoring on 11 December were family members (Fig 4).

#### Management of vaccinated contacts

This was the first time that EVD vaccination had been deployed as part of an outbreak response in Liberia. The integration of vaccination activities with routine response led to minor challenges, including difficulties ensuring that information was fully shared between the team doing the vaccination and the other parts of the response teams. Four vaccinated individuals developed symptoms, two of whom were contacts, both had field blood draws and tested negative for EVD (Table 1).

### Identification of missing contacts

At the start of the monitoring period, 29 contacts were missing; however, all were successfully located and classified using the methods described above. One of the missing contacts travelled to Rivercess County prior to initiation of contact tracing. MCHT informed Rivercess County Health Team of the contact. A general Community Health Volunteer (gCHV) found this missing contact when she gave birth at a local clinic. The contact and her newborn subsequently returned to Montserrado County and were monitored twice daily at home. All other missing contacts were located within Monrovia.

### Data management

The new dynamic contact tracing dashboard was developed mid-response so teams were unable to collect full household data for each contact, which would have been important had any contact become a case.

Competing priorities created difficulties for effective data management. The county data team was maintaining day-to-day responsibilities as well as those required for outbreak response without increasing human resources or training. This led to an over-reliance on partners for data management support, as well as unsustainable working hours leading to fatigue of the team and potentially impacting the quality control of data.

The provision of support to quarantined contacts required robust information sharing between the contact tracing teams and the partners providing this support. In the initial stages of the response, this information sharing was incomplete, partly due to a lack of clear terms of reference for each response pillar, resulting in incomplete delivery of food and other support items/services. The adapted data management procedures eventually supported good information sharing and effective support of persons in quarantine.

## Discussion

Delayed and ineffective contact tracing contributed to the extensive transmission of EVD during the 2014–2015 outbreak [16]. Clusters of EVD are likely to reemerge [13], therefore understanding and addressing the challenges of implementing and managing contact tracing remains essential to halting transmission and minimizing morbidity and mortality. Our analysis of contact tracing activities implemented in response to the Duport Road cluster of EVD built on methods first used in Margibi cluster (May 2015) [16] and is applicable to contact tracing for future Ebola outbreaks and outbreaks of other infectious diseases.

The index case and two other ill family members were identified and isolated quickly, and there was no transmission of EVD to other contacts. All identified contacts were located and completed monitoring, suggesting that the adaptations employed during the Duport Road response improved contact tracing effectiveness and should be considered for future responses.

Contact tracing includes extended periods of personal interactions during times of high stress and fear. There is subtlety and diplomacy required to become proficient at the activity that can be hard to gain from an initial classroom training. Utilizing experienced and proven contact tracers provided mentorship in this area and in basic job responsibilities for new tracers, improving overall tracing proficiency from the first day of response. Challenges and capacity gaps still existed but we believe they were mitigated by this practice.

Precautionary quarantine of contacts at home or in a guest house allowed contact tracers and supportive teams to very quickly identify any contacts who developed symptoms. While we did not randomize contacts to different interventions, we believe that the support services provided by the MOH and partners were instrumental in ensuring adherence to quarantine by providing support to contacts (food, water, and essential supplies). Enhanced cooperation between contact tracing teams and officials within the MOH facilitated supportive documentation for employers of contacts so the contacts did not fear job loss and were allowed to return to work after 21-day monitoring period. Importantly, the decision to provide these services led to a need for very substantial coordination, and there was a need to enhance data sharing procedures to allow this. There is also a potential downside of providing these services—we believe that the provision of services contributed to the fact that some persons who were not in fact contacts were listed and monitored.

Complete and rapid testing for all symptomatic contacts is critical to effective control of EVD clusters. All of the symptomatic contacts identified in the Duport Road response were rapidly tested. One factor that may have contributed to the completeness of this testing was the use of mobile laboratory teams to draw blood in the field for persons who were symptomatic but did not meet the EVD case definition, as it may have reduced the stress by eliminating the need to go to the ETU. This method differed from standard guidance of hospitalizing in strict isolation any contacts who developed symptoms^6^ and it could have resulted in additional exposures in the home if any of these ill contacts actually had EVD, or in the delay of care for underlying medical issues in those without EVD. The use of field-based testing needs to be based on a balanced consideration of the risks and benefits of such an approach.

The implementation of school surveillance facilitated additional community monitoring. Since none of the students where school-based surveillance was conducted developed EVD, it is not possible to draw conclusions about the school based approach to monitoring; we did find that the approach was feasible and acceptable, and would support its use as an approach to active case finding for a group of low-risk individuals.

Although the new contact tracing dashboard tool was implemented part way through the response, it allowed for visualization of contact tracing data over time, and facilitated communication among response team members. The contact tracing team’s regular collaboration with active case finding teams and case investigation teams resulted in the detection of previously unidentified contacts and the locations of missing contacts. When known contacts could not be found, the MOH’s collaboration with phone companies resulted in the location and follow-up of all known contacts.

### Recommendations

From experiences and lessons learned through the contact tracing activities for the Duport Road EVD outbreak, we recommend the following activities be implemented (Table 3).

**Table 3:** Key recommendations from the contact tracing activities of the Duport Road EVD outbreak, Montserrado County, November–December 2015.

Recommendations	Requirements
1) Clearly identify key personnel; include these individuals in county level emergency preparedness and response plan	Registry of trained contact tracers maintained at county level
	Regular refresher trainings for contact tracers and case investigators
	Simulation exercises
2) Develop standard operating procedures and job aids for contact tracing	Guidance documents for field use
	Communication and information sharing SOPs
	Data management protocols
3) Develop rapid response packages for contact tracers to reduce delay in deployment	Necessary supplies and protocols
	Response packages stored and stockpiled at county health offices
4) Create and implement data sharing procedures	Clear data sharing agreements at national and county levels
	Secure data-sharing platform
	Privacy and reporting trainings for journalists
5) Improve communication and coordination among response teams	Clear terms of reference for each response pillar
	Daily coordination meetings of the incident management system
	Strong coordination of partners
	Engagement of community leaders

First, needed personnel (regular and surge staff) should be clearly identified and included in the county level epidemic preparedness and response (EPR) plan. All of these staff should receive regular refresher training, including knowing acceptable temperature ranges for contacts under monitoring, proper use of Thermoflash thermometers, correct daily recording of monitoring information, reporting flow, and protocols. Refresher training should include simulation exercises with mentorship from experienced contact tracers. These trainings should address any fears and concerns among contact tracers so they know how to protect themselves while conducting monitoring activities. The county health teams should maintain a register of trained contact tracers with the date of the most recent refresher training completed so they can be used for future responses to EVD or other epidemic-prone diseases. Appropriate training and supervision of case investigators is also required to ensure only true contacts are listed and monitored.

Second, we recommend development of contact tracing procedures and job aides for field use and data management. These should be clear and concise documents that contact tracers and supervisors can take into the field with them for guidance. These procedures should include specific information about sharing of information between contact tracing teams and teams conducting vaccination activities. The data management procedures should explain best practices in contact tracing data management and detail how to use all the tools developed as a result of this outbreak, including the contact tracing data management dashboard.

The third recommendation is the development of a package for contact tracers that consists of all the necessary supplies and can be provided at the beginning of an outbreak to reduce the delay in deployment. When combined with regular refresher trainings, and a comprehensive set of guidance documents, this will enable rapid deployment of contact tracers.

The fourth recommendation is to ensure that procedures for data sharing are in place before responses are needed. These procedures should include development of clear data sharing agreements at county level and the national level, as well as between the county heath team and supporting partners. A pre-existing data sharing agreement will allow for immediate collaboration between those responding to an event of public health concern. We also recommend the development of a secure data sharing platform to protect confidential patient information. Procedures for sharing information with the press should also be developed. It may be useful to establish regulations and/or implement training about privacy for journalists.

The final recommendation is to focus on the improvement of communication and coordination to ensure that all teams are aware of the needs on the ground so they can respond accordingly, and to mitigate logistical challenges as they arise. As well as daily IMS meetings there is a need for clear terms of reference for each response pillar together with an organization chart stating lead persons and their contact details for each sector, this would need to be developed at the beginning of a response. Past outbreaks have demonstrated the need for strong coordination of partners and the engagement of community leaders to end EVD transmission [17].

The Duport Road response offered an opportunity to improve contact tracing methods employed in response to Ebola clusters in Liberia. The likelihood of EVD reemergence is high [3,5,17] and EVD remains a threat to the region. Therefore, prompt identification and monitoring of contacts remains one of the key actions necessary for ending the transmission of EVD.

## Supporting information

S1 Checklist STROBE checklist. (DOCX)
